# Liver failure as the initial presentation in cancer of unknown primary: a case report

**DOI:** 10.1186/s12879-023-08274-0

**Published:** 2023-05-30

**Authors:** Lisha Qin, Shan Tian, Lian Yang, Jun Fan, Jianchu Zhang

**Affiliations:** 1 Department of Respiratory and Critical Care Medicine, Union Hospital, Tongji Medical College, Huazhong University of Science and Technology, Wuhan, 430022 China; 2 Department of Infectious Diseases, Union Hospital, Tongji Medical College, Huazhong University of Science and Technology, Wuhan, 430022 China; 3 Department of Radiology, Union Hospital, Tongji Medical College, Huazhong University of Science and Technology, Wuhan, 430022 China; 4 Department of Pathology, Union Hospital, Tongji Medical College, Huazhong University of Science and Technology, Wuhan, 430022 China

**Keywords:** Liver failure, cancer of unknown primary, Malignant infiltration, Polymyositis, Case report

## Abstract

**Background:**

Liver failure is severe hepatic cellular damage caused by multiple factors that leads to clinical manifestations. Hepatic infiltration by malignancy is rarely reported as a cause of liver failure.

**Case presentation:**

A 51-year-old male patient was admitted to the Wuhan Union Hospital complaining of bloating and jaundice. He had been diagnosed with polymyositis ten prior and was taking oral glucocorticoids. Physical examination revealed seroperitoneum and icteric sclera; laboratory tests revealed liver dysfunction, a coagulopathy, and negative results for the common causes of liver failure. Moreover, an ascitic tap and bone marrow aspirate and trephine confirmed a metastatic, poorly differentiated adenocarcinoma. These findings indicate that malignant infiltration is the most likely cause of liver failure. Regrettably, the patient refused complete liver and lymph node biopsies and was discharged on day 31.

**Conclusion:**

Clinicians should consider the possibility of malignant infiltration when approaching a case of liver failure with prodromal symptoms or imaging abnormalities, especially in patients with autoimmune diseases, such as polymyositis.

**Supplementary Information:**

The online version contains supplementary material available at 10.1186/s12879-023-08274-0.

## Introduction

Liver failure is a condition in which previously healthy liver cells are extensively and severely damaged, resulting in the loss of essential liver functions, followed by the clinical manifestations of jaundice, coagulopathy, ascites, and hepatic encephalopathy [1]. Common causes of liver failure include drug-related liver injury, viral infections, hepatic ischemia, and autoimmune hepatitis [1, 2]. Moreover, a rare cause of liver failure that clinicians often overlook is malignant hepatic infiltration [3, 4]. Here, we report a rare case of liver failure, possibly secondary to hepatic infiltration by an adenocarcinoma; however, the primary site of the cancer has not been identified.

## Case report

A 51-year-old male patient presented to the Department of Infectious Diseases, Wuhan Union Hospital, on December 9, 2021, complaining of persistent abdominal distension and jaundice of unknown cause for approximately 30 days. He had visited a local hospital and the initial examination revealed liver dysfunction and ascites. However, liver-sparing and symptomatic treatment provided no symptom relief, and he visited the gastrointestinal surgery department of Wuhan Union Hospital for further treatment. During hospitalization, dynamic monitoring of laboratory indicators suggested progressive liver function deterioration. Thus, extensive examinations were performed to explore the possible causes, and revealed elevated tumor markers, while the viral serology (cytomegalovirus, Epstein-Barr virus, and hepatitis viruses), autoantibody profile, parasite, ceruloplasmin, and alpha-1 antitrypsin were negative (Figure S1 and Table S1).

Furthermore, imaging was completed, and magnetic resonance imaging of the liver revealed liver injury, portal hypertension, ascites, and esophageal and gastric varices (Figure S2). The 68Ga-FAPI PET and 18FDG-PET/ computed tomography (CT) yielded findings suggestive of metastases in bone (skull, ribs, cone, scapula, sacrum, ilium, pubis, and ischium) and lymph nodes (perigastric lymph nodes, retroperitoneal lymph nodes, mesenteric lymph nodes, and left supraclavicular lymph nodes) (Figure S3). Chest CT revealed enlarged lymph nodes in the mediastinum and hilum, considered to be lymphadenitis (Figure S4). Upper endoscopy and colonoscopy were also performed, no apparent signs of cancer (Figure S5). Given that the patient had no indications for surgery and no significant relief of symptoms after treatment, he was transferred to the Infection Diseases department on day 9. Ten years prior, he had been admitted with symmetrical, proximal weakness of the upper and lower extremities. Blood investigations revealed a markedly elevated creatine kinase, and electromyography and muscle biopsy demonstrated myopathy. After excluding other diseases, he had been diagnosed with polymyositis and treated with oral glucocorticoids. The patient reported that over the preceding ten years, he had visited a local hospital, taken medication regularly, and had adequate disease control, but no detailed medical records were available. He denied a history of hepatitis or alcohol consumption. Informed consent was obtained from the patient, and the requirement for ethical approval was waived by the research ethics committee of Wuhan Union Hospital because of the retrospective and anonymous nature of the study.

**Fig. 1:**
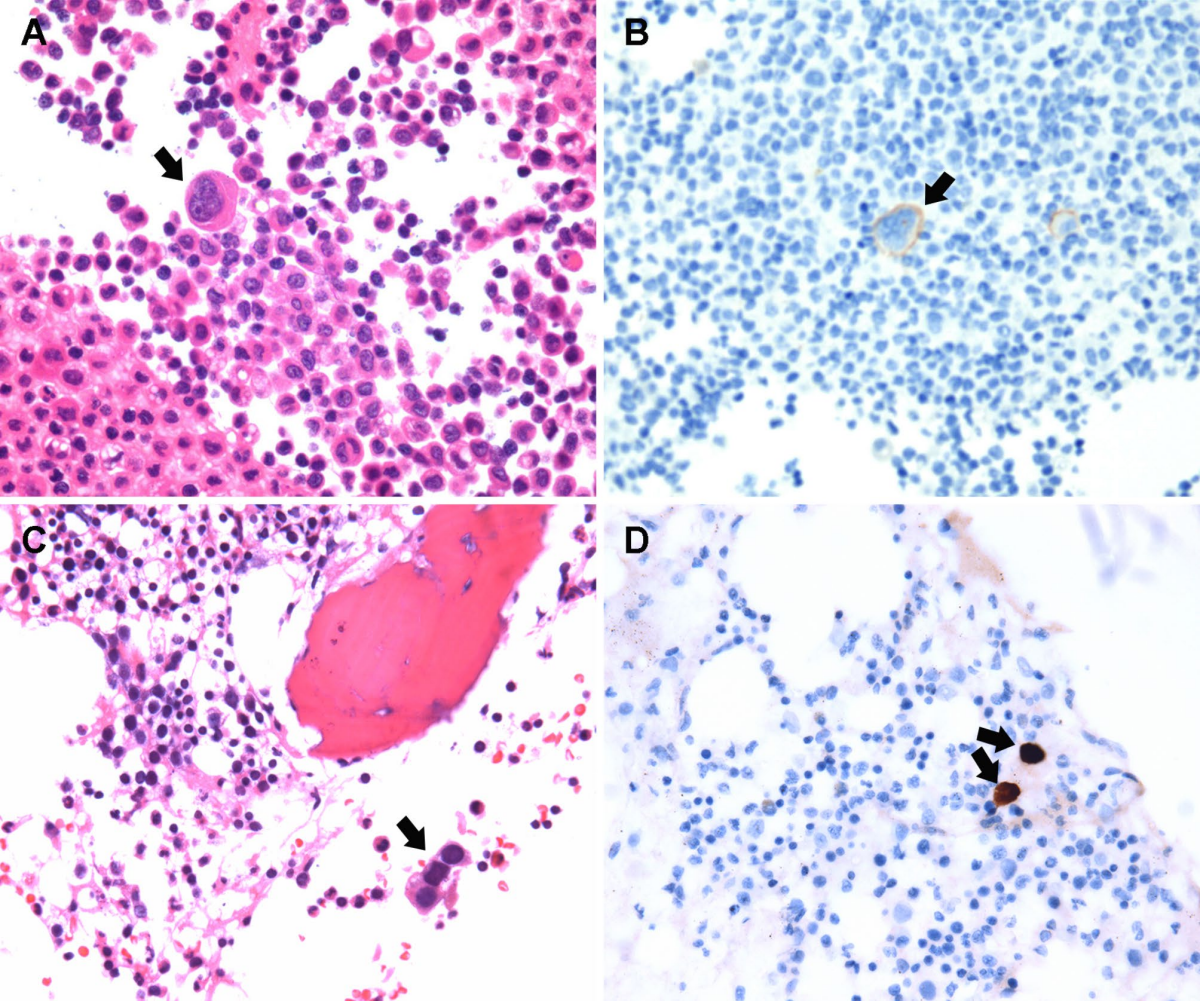
Ascites cytology and bone marrow biopsy histopathology. (A) Hematoxylin and eosin staining of ascites showed cancer cells (hematoxylin and eosin ×400). Positive immunocytochemical staining for BER-P4(+) (B), claudin-4(+), TTF1(+), CK7(+). (C) Bone marrow biopsy showed normal bone marrow structure and few tumor cells, suggesting a metastatic poorly differentiated adenocarcinoma (hematoxylin and eosin ×600). Positive immunohistochemistry staining for TTF-1 (D), PCK (+), CK7(+), Napsin A(+) and Cyclin D1(+)

Laboratory results were consistent with liver failure, and admission values were as follows: thrombocytopenia, hypoalbuminemia, and elevated levels of alanine aminotransferases, aspartate aminotransferase, alkaline phosphatase, total bilirubin, direct bilirubin, international normalized ratio (INR) and a prolonged prothrombin time (Fig. 1 and Table S1). Furthermore, laboratory tests revealed elevated immunoglobulin G4 and inflammatory markers, including a high white blood cell count, C-reactive protein (CRP), and procalcitonin (PCT). Metagenomic next-generation sequencing (mNGS) of the peripheral blood detected cytomegalovirus and *Aspergillus niger* infections. (Table S1).

Simultaneously, an ultrasound-guided ascitic tap, bone marrow aspiration and trephine (BMAT) were performed to assess for the presence of cancer. The ascitic tap cytology and BMAT histology revealed a metastatic, poorly differentiated adenocarcinoma of pulmonary origin. Moreover, the immunohistochemical profile of the bone marrow trephine was positive for PCK, CK7, TTF1, Napsin A, and Cyclin D1, but negative for CK20, PAX8, CDX2, Villin, Syn, P63, CD3, CD20, and CD30 (Fig. 1). Furthermore, ascitic fluid analysis revealed the following: Clear, yellow fluid, a white blood cell count of 399 × 10^6^/L, 80.6% mononuclear cells, 4% multinucleated cells, an adenosine deaminase of 4.0 U/L, lactate dehydrogenase of 74 IU/L, and a serum ascites albumin gradient of 26.3 g/L (serum albumin 31.0 g/L, ascites albumin 4.7 g/L).

Considering the history of polymyositis and the newly discovered tumor, combined with extensive examinations, the primary diagnosis was liver failure, possibly secondary to malignant infiltration by a cancer of unknown primary origin, polymyositis, and/or infection. During hospitalization, the patient was treated with methionine and vitamin B1, hepatocyte growth-promoting factors, magnesium isoglycyrrhizinate, and reduced glutathione, which relieved the hepatitis through its effects on reducing enzymes and protecting hepatocytes. Additionally, the appropriate examinations were completed after consultation with a rheumatologist, and the medication was adjusted to total glucosides of paeony and hydroxychloroquine due to the history of polymyositis. Furthermore, antiviral (ganciclovir), antibacterial (Ceftriaxone Sodium and Tazobactam Sodium) and antifungal (Caspofungin Acetate) therapies were also added in response to the elevated CRP, PCT, and positive mNGS results. Moreover, this patient was administered diuretics, human gamma globulin, a plasma infusion, and albumin supplements to improve his symptoms. Regrettably, the patient refused further examination and treatment and was discharged on Day 31.

We followed-up on the patient one month after discharge and learned that he did not receive further treatment and died within one week of discharge.

## Discussion and conclusions

Although the liver is the most common site for hematogenous metastasis of malignant solid tumors, clinically severe hepatic dysfunction is rare. Furthermore, diagnosing liver failure secondary to malignancy can be challenging, not only because of nonspecific symptoms and worthless standard laboratory values, but also because the imaging of the liver may be notable for the absence of large mass lesions, which may initially appear normal or as “pseudocirrhosis.” The definitive diagnosis is only confirmed by liver biopsy or autopsy [5, 6]. Unfortunately, the patient refused to undergo a liver biopsy. Therefore, whether a malignant tumor played a critical role in liver failure in this case still needed further investigation.

The most common malignant liver infiltrates are hematologic malignancies, followed by breast and gastrointestinal cancer [7]. A few scattered reports also identified metastatic carcinoma from the lungs [6, 8–10], most commonly small cell lung cancers, contrary to our current findings. In this case, the pathologic examination of the ascitic fluid and bone marrow biopsy suggested that lung adenocarcinoma was the most likely histopathological type, even though a primary lung lesion was not identified. Furthermore, the prognosis is poor in most patients with liver failure secondary to malignant infiltration. Deciding on the ideal time to start treatment and which treatment regimen to use is difficult. This patient eventually refused treatment and was discharged on day 31 of admission. This also suggests that clinicians should perform thorough evaluations to identify unusual etiologies, especially in patients with liver failure complicated by autoimmune diseases. Successful diagnosis and timely treatment may improve patient prognoses to a certain extent.

Recent studies have revealed that cytomegalovirus causes or contributes to liver failure [11, 12]. In this case, the virology study of cytomegalovirus yielded negative results, while cytomegalovirus was detected in the mNGS of the peripheral blood. However, the effects of this infection on the patient’s disease progression remain unknown. A retrospective case-control study indicated that cytomegalovirus activation might significantly increase mortality in patients with liver failure [12]. Therefore, early identification and treatment planning may improve liver failure prognosis, and timely antiviral treatment should be administered to such patients.

This patient had a 10-year history of polymyositis. Previous studies have shown that polymyositis is associated with an increased risk of malignant disease, which may occur before, concurrent with, or after the diagnosis of polymyositis [13, 14]. In this case, the cancer was discovered ten years after polymyositis was diagnosed. Moreover, the multi-site pathology of this patient suggested that the tumor most likely originated from the lung, neither PET/CT nor chest CT revealed signs of lung cancer, and only an enlarged mediastinal lymph node was identified as lymphadenitis. Furthermore, the patient exhibited no respiratory symptoms throughout the disease course. Failure to detect the primary site may be related to not only the small size, but also the involvement of immunologic mechanisms leading to the spontaneous regression of the primary lesion [15]. Therefore, clinicians must perform a comprehensive and systematic screening to rule out tumors if a patient with polymyositis presents with symptoms unrelated to polymyositis in the course of the disease.

According to the latest guidelines, the diagnosis of liver cirrhosis can be established by the following: chronic liver disease, elevated serum bilirubin, prolongation of the INR, and single or multiple organ failure [16]. After the patient was admitted to the hospital, imaging and laboratory tests were performed. Combined with multiple test results, viral hepatitis, autoimmune hepatitis, alcoholic hepatitis, parasitic infections, and genetic and metabolic disease-related hepatitis were ruled out. Moreover, a diagnosis of chronic hepatitis and liver failure was established. However, we believe that the patient’s liver failure might have resulted from a combination of multiple factors, including but not limited to chronic hepatitis, tumor liver infiltration, and drug factors.

Liver failure management is based on the treating precipitant events and supportive therapy for organ failure [16]. More specifically, empirical broad-spectrum antimicrobials should be promptly initiated when an infection is suspected. Preventing drug-induced liver injury and treatment of chronic liver diseases, such as alcoholic hepatitis and viral hepatitis B are necessary. Supportive treatment included nutritional support, albumin supplementation, plasma exchange, and granulocyte colony-stimulating factor. Liver transplantation is an effective treatment option for patients with liver failure.

In conclusion, for every case of liver failure with prodromal symptoms or imaging abnormalities, malignant infiltration should be considered in addition to common causes, especially in patients with autoimmune diseases, such as polymyositis.

## Electronic supplementary material

Below is the link to the electronic supplementary material.

Supplementary Material 1

Supplementary Material 2

Supplementary Material 3

Supplementary Material 4

Supplementary Material 5

Supplementary Material 6

Supplementary Material 7

## Data Availability

All data and materials are available with the first author.

## Abbreviations

CT: Computed tomography
INR: International normalized ratio
CRP: C-reactive protein
PCT: Procalcitonin
mNGS: Metagenomic next-generation sequencing
BMAT: Bone marrow aspiration and trephine
