# Supplementary material for: Liver failure as the initial presentation in cancer of unknown primary: a case report
Source: BMC Infect Dis. 2023 May 30;23:363. doi: 10.1186/s12879-023-08274-0 (PMC10228056; doi:10.1186/s12879-023-08274-0)
Supplement: Supplementary file 1 — Supplementary Material 1 [file 12879_2023_8274_MOESM1_ESM.pdf]

CARE Checklist of information to include when writing a case report

(cc) BY-NC-ND

Topic	Item	Checklist item description	Reported on Line
Title	1	The diagnosis or intervention of primary focus followed by the words "case report"	Title, page1
Key Words	2	2 to 5 key words that identify diagnoses or interventions in this case report, including "case report"	Key words, page2
Abstract (no references)	3a	Introduction: What is unique about this case and what does it add to the scientific literature?	Abstract, page2
	3b	Main symptoms and/or important clinical findings	Abstract, page2
	3c	The main diagnoses, therapeutic interventions, and outcomes	Abstract ,page2
	3d	Conclusion—What is the main "take-away" lesson(s) from this case?	Abstract ,page2
Introduction	4	One or two paragraphs summarizing why this case is unique (**may include references**)	Introduction, page3
Patient Information	5a	De-identified patient specific information.	Case presentation, paragraph 1
	5b	Primary concerns and symptoms of the patient.	Case presentation, paragraph 1
	5c	Medical, family, and psycho-social history including relevant genetic information	Case presentation, paragraph 2
	5d	Relevant past interventions with outcomes	Case presentation, paragraph1
Clinical Findings	6	Describe significant physical examination (PE) and important clinical findings.	Case presentation, paragraph1- 4
Timeline	7	Historical and current information from this episode of care organized as a timeline	Not applicable
Diagnostic Assessment	8a	Diagnostic testing (such as PE, laboratory testing, imaging, surveys).	Case presentation, paragraph 1- 4
	8b	Diagnostic challenges (such as access to testing, financial, or cultural)	Case presentation, paragraph 5
	8c	Diagnosis (including other diagnoses considered)	Case presentation, paragraph 5
	8d	Prognosis (such as staging in oncology) where applicable	Not applicable
Therapeutic Intervention	9a	Types of therapeutic intervention (such as pharmacologic, surgical, preventive, self-care)	Case presentation, paragraph 5
	9b	Administration of therapeutic intervention (such as dosage, strength, duration)	Not applicable
	9c	Changes in therapeutic intervention (with rationale)	Case presentation, paragraph 5
Follow-up and Outcomes	10a	Clinician and patient-assessed outcomes (if available)	Not applicable
	10b	Important follow-up diagnostic and other test results	Not applicable
	10c	Intervention adherence and tolerability (How was this assessed?)	Not applicable
	10d	Adverse and unanticipated events	Case presentation, paragraph 5
Discussion	11a	A scientific discussion of the strengths AND limitations associated with this case report	Not applicable
	11b	Discussion of the relevant medical literature **with references**.	Discussion and conclusion Paragraph1- 3
	11c	The scientific rationale for any conclusions (including assessment of possible causes)	Discussion and conclusion Paragraph1- 3
	11d	The primary "take-away" lessons of this case report (without references) in a one paragraph conclusion	Discussion and conclusion Paragraph4
Patient Perspective	12	The patient should share their perspective in one to two paragraphs on the treatment(s) they received	Not applicable
Informed Consent	13	Did the patient give informed consent? Please provide if requested	Yes ☐ No ☑
