# Supplementary material for: Liver failure as the initial presentation in cancer of unknown primary: a case report
Source: BMC Infect Dis. 2023 May 30;23:363. doi: 10.1186/s12879-023-08274-0 (PMC10228056; doi:10.1186/s12879-023-08274-0)
Supplement: Supplementary file 2 — Supplementary Material 2 [file 12879_2023_8274_MOESM2_ESM.pdf]

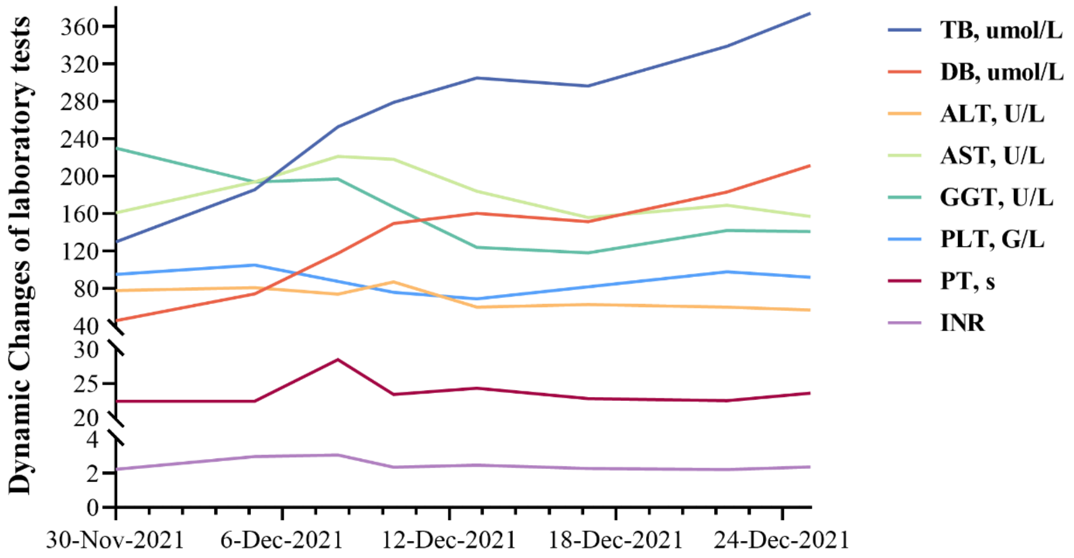

Figure S1. Dynamic changes of laboratory tests.

PLT: platelets, TB: total bilirubin, DB: direct bilirubin, ALT: alanine aminotransferases, AST: aspartate aminotransferase, PT: prothrombin time, INR: international normalized ratio.
