# Supplementary material for: Liver failure as the initial presentation in cancer of unknown primary: a case report
Source: BMC Infect Dis. 2023 May 30;23:363. doi: 10.1186/s12879-023-08274-0 (PMC10228056; doi:10.1186/s12879-023-08274-0)
Supplement: Supplementary file 3 — Supplementary Material 3 [file 12879_2023_8274_MOESM3_ESM.pdf]

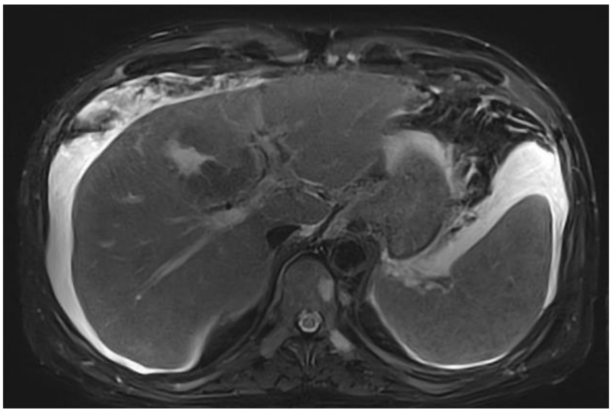

Figure S2. Magnetic resonance imaging (MRI) of the Liver.

The results revealed a diffuse decreased uptake of the liver parenchyma.
