# Supplementary material for: Liver failure as the initial presentation in cancer of unknown primary: a case report
Source: BMC Infect Dis. 2023 May 30;23:363. doi: 10.1186/s12879-023-08274-0 (PMC10228056; doi:10.1186/s12879-023-08274-0)
Supplement: Supplementary file 4 — Supplementary Material 4 [file 12879_2023_8274_MOESM4_ESM.pdf]

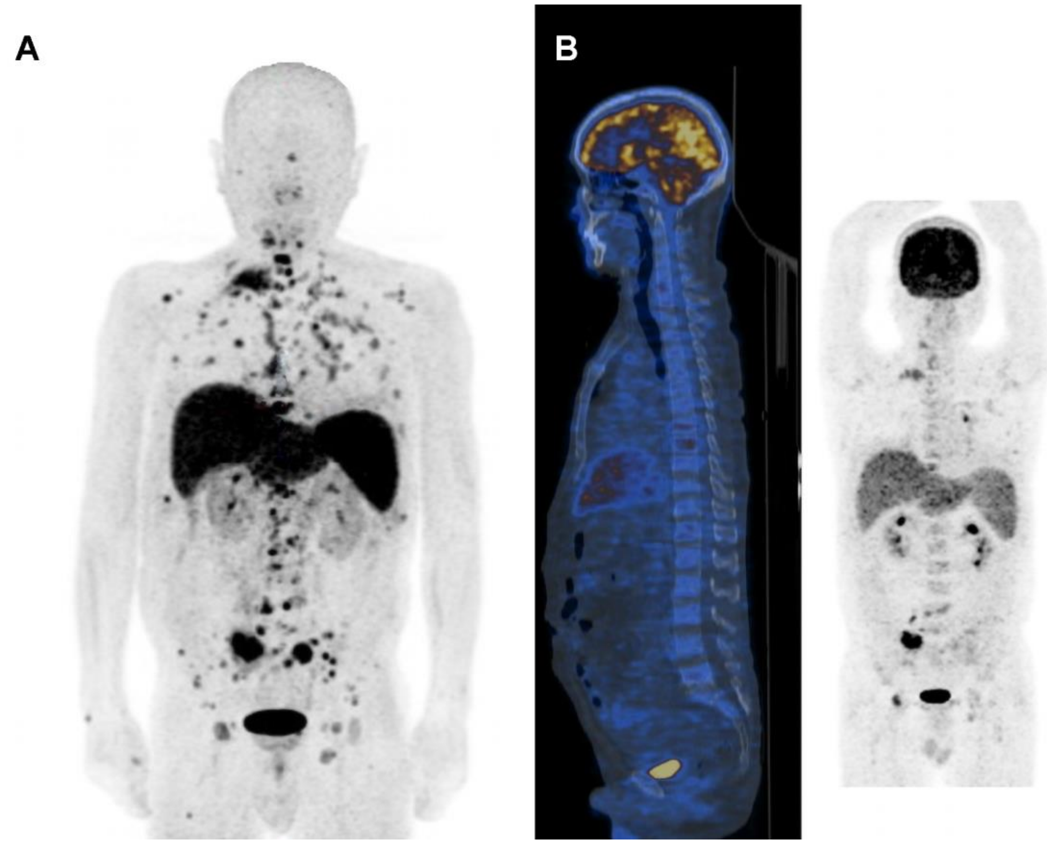

Figure S3. The 68Ga-FAPI PET and 18FDG-PET/CT imaging.

(A) Multiple areas of increased FAPI uptake were observed in the bone and lymph nodes, with SUVmax values of 2.5-14.6 and 8.0, respectively. These manifestations above cannot be ruled out as metastasis disease. Lymph nodes scattered in the mediastinum and the left hilar region with SUVmax 4.7-7.5 were due to lymphadenitis. In addition, diffuse FAPI uptake was detected in the liver and spleen. (B) Intensive uptake in multiple bones indicates metastatic lesions. Moreover, abnormal uptake was observed in the spleen and mediastinal lymph node.
