# Supplementary material for: Liver failure as the initial presentation in cancer of unknown primary: a case report
Source: BMC Infect Dis. 2023 May 30;23:363. doi: 10.1186/s12879-023-08274-0 (PMC10228056; doi:10.1186/s12879-023-08274-0)
Supplement: Supplementary file 5 — Supplementary Material 5 [file 12879_2023_8274_MOESM5_ESM.pdf]

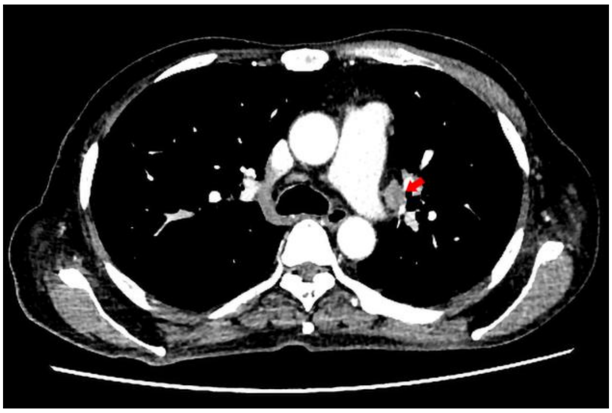

Figure S4. Lung imaging findings

Chest computed Tomography revealed increased and enlarged lymph nodes in the mediastinum and bilateral hila.
