# Supplementary material for: Liver failure as the initial presentation in cancer of unknown primary: a case report
Source: BMC Infect Dis. 2023 May 30;23:363. doi: 10.1186/s12879-023-08274-0 (PMC10228056; doi:10.1186/s12879-023-08274-0)
Supplement: Supplementary file 6 — Supplementary Material 6 [file 12879_2023_8274_MOESM6_ESM.pdf]

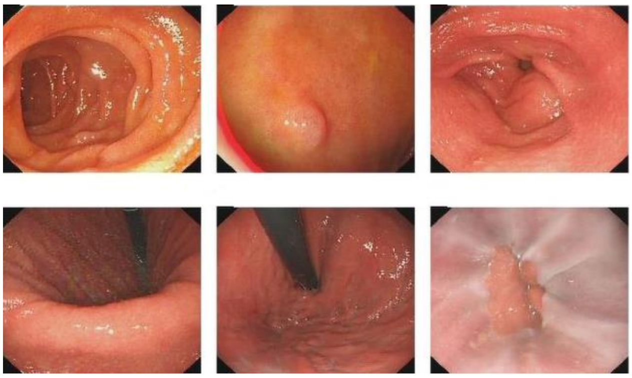

Figure S5. Gastroscopy findings

Upper gastrointestinal endoscopy revealed no varices in the esophagus or the gastric fundus. Meanwhile, the presence of strip redness of the gastric mucosa, diffuse redness of the gastric antrum mucosa, and polyps of the duodenal bulb were observed on endoscopy.
