# Supplementary material for: Liver failure as the initial presentation in cancer of unknown primary: a case report
Source: BMC Infect Dis. 2023 May 30;23:363. doi: 10.1186/s12879-023-08274-0 (PMC10228056; doi:10.1186/s12879-023-08274-0)
Supplement: Supplementary file 7 — Supplementary Material 7 [file 12879_2023_8274_MOESM7_ESM.docx]

**Table S1: Dynamic changes and positive results of laboratory tests**

| **Indexes** | **2021/11/30** | **12/5** | **12/8** | **12/10** | **12/13** | **12/17** | **12/22** | **12/25** |
| --- | --- | --- | --- | --- | --- | --- | --- | --- |
| **WBC, 10^9 /L** | 8.42 | 6.87 |  | 12.61 | 9.78 |  | 12.54 | 13.31 |
| **PLT, 10^9 /L** | 95 | 105 |  | 76 | 69 |  | 98 | 92 |
| **TB, umol/L** | 129.7 | 185.7 | 252.7 | 278.7 | 304.8 | 296.2 | 338.7 | 373.9 |
| **DB, umol/L** | 45.7 | 74.3 | 117.5 | 149.5 | 160.2 | 151.5 | 183 | 211.3 |
| **ALT, U/L** | 78 | 81 | 74 | 87 | 60 | 63 | 60 | 57 |
| **AST, U/L** | 161 | 194 | 221 | 218 | 184 | 156 | 169 | 157 |
| **Albumin, g/L** | 28.2 | 27.8 | 30.2 | 31 | 29.7 | 29.9 | 31 |  |
| **ALP, U/L** | 574 | 667 | 676 | 695 | 535 | 618 | 717 | 803 |
| **GGT, U/L** | 230 | 194 | 197 | 167 | 124 | 118 | 142 | 141 |
| **PT, s** | 22.4 | 22.4 | 28.5 | 23.4 | 24.3 | 22.8 | 22.5 | 23.6 |
| **INR** | 2.2 | 3.0 | 3.1 | 2.4 | 2.5 | 2.3 | 2.2 | 2.4 |
| **CRP, mg/L** |  | 56.9 |  | 66.3 | 59.4 | 43.7 | 44.5 | 35.8 |
| **ANA** |  | ± |  |  |  |  |  |  |
| **Anti-hepatitis A virus IgG antibody** |  |  | 18.67(+) |  |  |  |  |  |
| **CEA，ug/L** |  |  |  | ＞1500 |  |  |  |  |
| **CA125, U/ml** |  |  |  | ＞1000 |  |  |  |  |
| **CA19-9, U/ml** |  |  |  | 53.9 |  |  |  |  |
| **CA15-3, U/ml** |  |  |  | 68.8 |  |  |  |  |
| **CA72-4, U/ml** |  |  |  | 73.96 |  |  |  |  |
| **CYFRA21-1, ng/ml** |  |  |  | 32.74 |  |  |  |  |
| **IgG4, g/l** |  |  |  | 2.850 |  |  |  |  |
| **NSE, ug/L** |  |  |  | 19.56 |  |  |  |  |
| **mNGS results of peripheral blood** |  |  |  |  |  | CMV, Aspergillus niger |  |  |

WBC: white blood cell, PLT: platelets, TB: total bilirubin, DB: direct bilirubin, ALT: alanine aminotransferases, AST: aspartate aminotransferase, ALP: alkaline phosphatase, GGT: gamma-glutamyl transferase, PT: prothrombin time, INR: international normalized ratio, CRP: C-reactive protein, ANA: antinuclear antibody, CEA: carcinoembryonic antigen, CA125: carbohydrate antigen 125, CA19-9: carbohydrate antigen19-9, CA15-3: carbohydrate antigen15-3, CA72-4: carbohydrate antigen72-4, IgG4: immunoglobulin G4, NSE: neuron-specific enolase, mNGS: metagenomics next generation sequencing.
